# Analysis of polygenic risk score usage and performance in diverse human populations

**DOI:** 10.1038/s41467-019-11112-0

**Published:** 2019-07-25

**Authors:** L. Duncan, H. Shen, B. Gelaye, J. Meijsen, K. Ressler, M. Feldman, R. Peterson, B. Domingue

**Affiliations:** 1 Department of Psychiatry and Behavioral Sciences, Stanford University, 401 Quarry Road, Stanford, CA 94305 USA; 2 Department of Epidemiology, Harvard T.H. Chan School of Public Health, 667 Huntington Ave, Kresge 505, Boston, MA 02115 USA; 3 Mailman Research Center, Harvard Medical School, McLean Hospital, 115 Mill St, Belmont, MA 02478 USA; 4 Department of Biology, Stanford University, Herrin 478A, Stanford, CA 94305 USA; 5 Department of Psychiatry, Virginia Institute for Psychiatric and Behavioral Genetics, Virginia Commonwealth University, P.O. Box 980003, Richmond, VA 23298 USA; 6 Graduate School of Education, CERAS 510, Stanford University, Stanford, CA 94305 USA

**Keywords:** Genome-wide association studies, Genetic variation, Predictive markers, Risk factors

## Abstract

A historical tendency to use European ancestry samples hinders medical genetics research, including the use of polygenic scores, which are individual-level metrics of genetic risk. We analyze the first decade of polygenic scoring studies (2008–2017, inclusive), and find that 67% of studies included exclusively European ancestry participants and another 19% included only East Asian ancestry participants. Only 3.8% of studies were among cohorts of African, Hispanic, or Indigenous peoples. We find that predictive performance of European ancestry-derived polygenic scores is lower in non-European ancestry samples (e.g. African ancestry samples: *t* = −5.97, *df* = 24, *p* = 3.7 × 10^−6^), and we demonstrate the effects of methodological choices in polygenic score distributions for worldwide populations. These findings highlight the need for improved treatment of linkage disequilibrium and variant frequencies when applying polygenic scoring to cohorts of non-European ancestry, and bolster the rationale for large-scale GWAS in diverse human populations.

## Abstract (web summary)

Predominant participation of European-ancestry individuals in genetic studies has hindered the better understanding of genetic risk in non-European ancestry individuals. Here, Duncan et al. quantify polygenic risk score use and performance in worldwide populations.

## Introduction

The over-representation of participants of European ancestry in human genetics research has been broadly acknowledged^1–5^, and increasing the representation of diverse populations has recently become a higher priority for the research community^5–10^. This has led funding agencies such as the National Institutes of Mental Health to prioritize genetic studies of diverse populations. Accordingly, representation of non-European ancestry participants in genome-wide association studies (GWAS) increased, from 4% in 2009^1^ to 19% in 2016^3^. Most of the increase in non-European ancestry research is attributable to expansion of genetic studies of East Asian populations, as reported previously^3^ and as observed in our data (see below). Thus, most populations are still severely under-represented. This lack of representation, if not mitigated, will limit our understanding of etiological factors predisposing to disease risk, and will hinder efforts to develop precision medicine. It is also important to understand the implications of the European-centric bias of earlier genetic studies for work that builds upon existing research. For example, researchers need to know how the limited diversity in earlier medical genetic studies impacts the use of polygenic risk scores in non-European ancestry populations.

The use of polygenic risk scores^11,12^ (PRS, also known as risk profile scoring, genetic scoring, and genetic risk scoring) has become widespread in biomedical and social science disciplines^13–15^. Businesses have commercialized this technology, including direct-to-consumer testing from 23andMe and other companies. Perhaps most importantly, there is hope that polygenic risk scores can improve health outcomes by accelerating diagnosis and matching patients to tailored treatments^16^. Polygenic scoring studies have demonstrated reliable, though modest, prediction using straightforward scoring methods^11,12^ for many complex genetic phenotypes (e.g., blood pressure^13,17^, height^18^, diabetes^9,19^, depression^7,20^, and schizophrenia^14^). Polygenic risk scores are calculated by summing risk alleles, which are weighted by effect sizes derived from GWAS results^11,12,21^. Commonly used methods account for ancestry using principal components (calculated on pruned genetic data). In the parlance of polygenic scoring studies, the training GWAS is referred to as the discovery sample, and the testing dataset is referred to as the target sample. No overlap between training and testing datasets is essential to maintain independence of predictions, and the removal of related individuals is also needed, as demonstrated by Wray et al.^21^. Methods of prediction that offer modest improvements on this basic framework are also available^22–25^.

Polygenic scores can be constructed for any complex genetic phenotype for which appropriate GWAS (or other robust association) results are available. The challenges inherent in using polygenic scores—including modest capacity for prediction and necessary considerations regarding statistical power—have been reviewed previously^21,26^. Recent research has focused on the generalizability of polygenic scores to non-European ancestry populations^27^. There is good reason to anticipate reduced predictive power in non-European ancestry samples because of differences in variant frequencies and linkage disequilibrium patterns between populations^12,28^. However, the magnitude of performance decrease is largely unknown. Few systematic studies of polygenic score performance across different ancestry groups are available, though see Hoffman et al.^13^ for an investigation of blood pressure metrics and a recent UK Biobank analysis^27^. Further, some previous findings may need to be re-evaluated in light of newer findings about relationships between ancestry and GWAS results^29–31^. Thus, there is a need for systematic evaluation of polygenic score performance across multiple populations, phenotypes, and samples.

In this manuscript we answer questions about the predictive performance of polygenic scores. Based on ancestry, we find large statistically significant differences in performance between populations. A second major area of inquiry concerns differences in the distributions of polygenic scores for worldwide populations, as currently calculated^30–39^. (The term “worldwide populations” is used in this manuscript. It is important to note that there are many meanings and operationalizations of the term “population”, and that only some of the world’s populations are represented in genetic analyses). Multiple potential causes of observed distribution differences of polygenic scores have been reported, including drift^32^, selection^33,36–39^, artifactual differences due to uncorrected population stratification^30,31^, and different environmental effects^40,41^. We show that investigator-driven choices in the construction of polygenic scores also significantly impacts distributions of these polygenic scores for worldwide populations. Finally, in contrast to putative claims about the effect (or lack thereof) of polygenic score differences on phenotypic differences among worldwide populations, we show that current knowledge is insufficient to either confirm or refute such reports. These results calibrate researcher expectations about polygenic score performance in different populations and demonstrate that treatment of ancestry depends critically on how polygenic scores are constructed, for diverse non-European ancestry populations.

## Results

### PRS usage and performance in worldwide populations

**Fig. 1:**
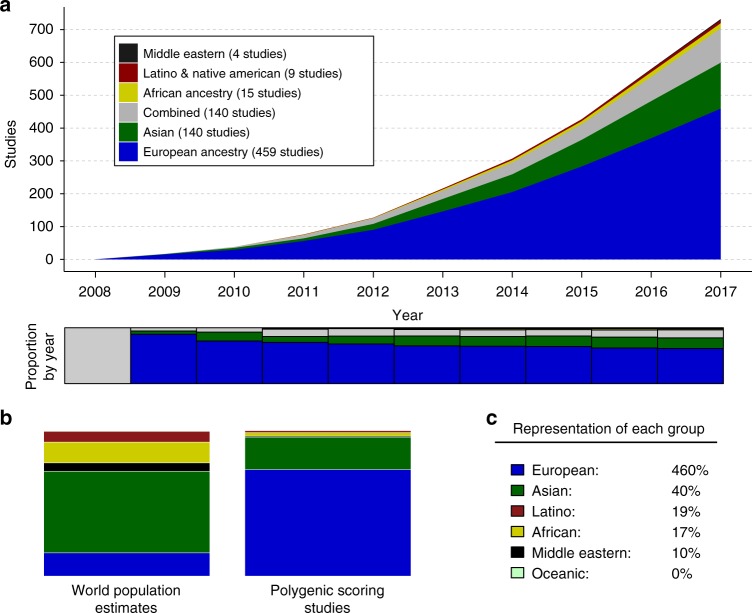
Ancestry representation in the first decade of polygenic scoring studies (2008–2017; *N* = 733 studies). **a** Cumulative numbers of studies by year are denoted by color. The stacked bar graph below the cumulative distribution plot shows proportional ancestry by year. **b** Stacked bar charts depict world ancestry representation (left) and polygenic scoring study representation (right). **c** The percentage representation for each ancestry group is given, such that 100% would indicate equal representation in the world and in polygenic scoring studies. For example, European ancestry samples are over-represented (460%) whereas African ancestry samples are under-represented (17%)

How well-different ancestry groups have been represented in the first decade of polygenic scoring research (2008–2017, inclusive) is shown in Fig. 1a, which presents cumulative distributions of numbers of studies for specific ancestry groups across time. The field has been dominated by European ancestry studies. Across the 733 studies examined (see Methods for inclusion criteria and Supplementary Data 1 for a list of studies), 67% included exclusively European ancestry participants. There have also been 140 studies conducted in exclusively Asian populations (19%), most commonly in East Asian countries (e.g., China and Japan). Only 3.8% of the polygenic studies from the first decade of polygenic scoring research concerned populations of African, Latino/Hispanic, or Indigenous peoples combined. (Note that we retain population names from the original reports (e.g., Native American and Middle Eastern) in Fig. 1 in order to maintain consistency in terminology. Combined denotes that more than one ancestry group was included in the study (e.g., European ancestry and Asian ancestry participants)). These results are similar to those reported by Popejoy and Fullerton^3^, who noted that non-European ancestry representation in GWASs was almost exclusively in Asian populations, and East Asian populations in particular.

By comparing representation of particular ancestry groups with world population estimates for those groups (Fig. 1b), it is possible to quantify the over- or under-representation of each major ancestry group. European ancestry representation was ~460% of what it would be if representation was proportional to world ancestry. In contrast, African ancestry (17%) and Latino samples (19%) were under-represented relative to world populations. East and South Asian samples are combined in this figure, but it should be noted that representation of East Asian samples is much higher than South Asian samples, which have been included in very few polygenic scoring studies to date. Relative to world populations for these groups, Middle Eastern and Oceanic populations have the lowest representation in polygenic scoring studies (10% and 0%, respectively).

**Fig. 2:**
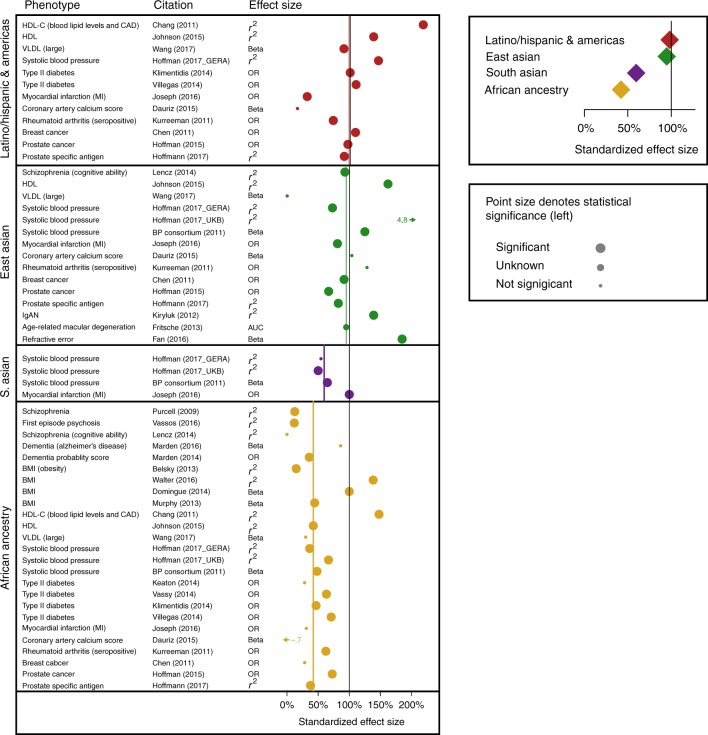
Forest plot of performance shows variation in polygenic score performance by ancestry (26 studies). Each row in the forest plot (left) represents one pair of polygenic analyses (i.e., in a non-European ancestry sample and a matched European ancestry sample from the same study. Phenotypes, citation information, and available effect sizes are given for each comparison. The vertical black line at 100% corresponds to equal performance in the non-European ancestry and the European ancestry samples. Vertical colored lines denote median standardized effect sizes, for each of the major ancestry groups. On the top right, median values for standardized effect sizes, for each major ancestry group, are given. Standard errors are not provided because many studies lacked sufficient information; however, statistical significance of each non-European ancestry analyses is denoted by point size. HDL-C high density lipoprotein cholesterol, VLDL very low-density lipoprotein, GERA Genetic Epidemiology Research on Aging, OR odds ratio, UKB UK Biobank, IgAN immunoglobulin A nephropathy, AUC area under the curve, BP blood pressure, BMI body mass index

Having analyzed the use of polygenic scores in different ancestry groups (above), we next assessed the performance of polygenic scores in multiple ancestry groups. Since most large-scale GWAS have been conducted in primarily (or exclusively) European ancestry individuals^42^, our a priori hypothesis was that polygenic scores would perform best among European ancestry individuals, and less well for other populations. Figure 2 provides an overview of polygenic score performance across ancestry groups. Results from all complex genetic phenotypes are analyzed together in order to increase the amount of data available for analysis. In Fig. 2, each point represents one within-study comparison between a non-European ancestry sample and the matched (within-study) European ancestry sample. The vertical black line represents equal performance in the non-European ancestry sample, as compared to the matched European ancestry sample from the same study.

As shown in Fig. 2, polygenic score performance was worst among African ancestry samples. The median effect size of polygenic scores in African ancestry samples was only 42% that of matched European ancestry samples (*t* = −5.97, *df* = 24, *p* = 3.7 × 10^−6^). Relative to matched European ancestry samples, performance was also lower in South (60%) and East Asian (95%) samples, but not significantly so (see top right portion of Fig. 2). In sum, an expectation of poorer polygenic score performance in non-European ancestry populations seems reasonable given these data. Attenuation of predictive performances is likely to be most extreme in samples of African ancestry, consistent with, on average, greater genetic distance between European and African ancestry populations, than between European and other ancestry populations^28,43^.

### Methodological choices impact polygenic score distributions

**Fig. 3:**
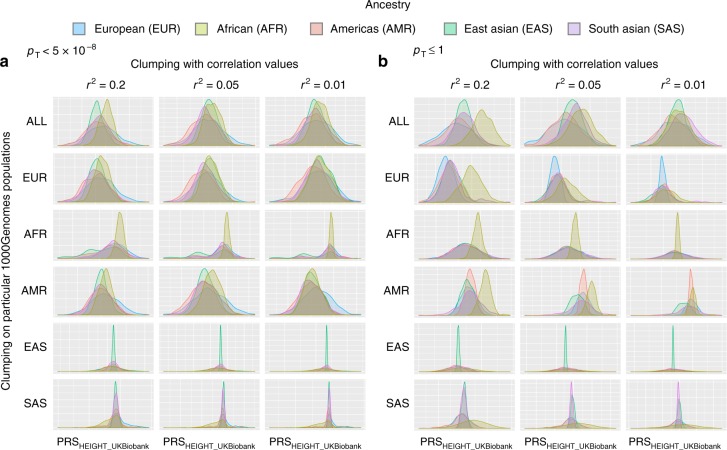
Polygenic score distributions vary by ancestry and methodical choices. For polygenic score construction, clumping is often used, and investigator-driven choices can produce large differences in score distributions for global populations. Polygenic score distributions for the five major 1000Genomes populations are plotted, showing how investigator-driven choices impact score distributions. For all plots, weights were derived from the UK biobank height GWAS. Both *r*^2^ values used in clumping (*r*^2^ *=* .2, .05, .01; see columns) and 1000Genomes populations used for clumping were varied (ALL, EUR, AFR, AMR, EAS, SAS; see rows). **a**, **b** correspond to the *p*-value threshold (*p*_T_) applied to the height summary statistics. **a** *p*_T_ = genome-wide significant variants (*p* < 5 × 10^−8^); **b** *p*_T_ = full genome variants (*p* < 1). PRS=polygenic risk score. ALL union of five 1000Genomes populations

We now consider questions about possible differences in polygenic scores among ancestral populations. Polygenic scores, as currently calculated, vary with ancestry. Indeed, polygenic scoring practices from as early as 2009 accounted for this^12^. The method used by Purcell et al. in 2009^12^ (and frequently since) includes two steps for mixed ancestry samples. First, samples are separated into more ancestrally homogeneous subgroups (using visual inspection of plots of principal components calculated on all genetic data from all samples). Second, principal components are calculated again within each of these more ancestrally homogeneous subgroups, and are used as covariates in polygenic scoring analyses, which are conducted separately within each subgroup. However, some research groups are not aware of these methodological recommendations and others (understandably) prefer a more inclusive analytical approach of analyzing all samples together (instead of creating subgroups). Figure 3 demonstrates why care must be taken in treatment of ancestry in polygenic scoring studies.

As shown in Fig. 3, methodological choices in the construction of polygenic risk scores can cause dramatic differences in distributions of polygenic risk scores for worldwide populations (polygenic scores were constructed for all 1000Genomes participants, *N* = 2577, see Methods for additional details). In general, the inclusion of more variants caused greater dispersion of distributions for these 1000Genomes populations (i.e., comparing panel A with panel B; genome-wide significant variants to all variants). Lower *r*^2^ thresholds for clumping tended to make worldwide population distributions more similar (left to right, in both A and B), and use of particular populations for clumping also dramatically affected distributions, particularly when East and South Asian populations were used for clumping. For further demonstration of the large effect that these methodological choices have on polygenic score distributions, see Supplementary Fig. 1 (polygenic scores for height^18^, weighted with GIANT) and Supplementary Fig. 2 (polygenic scores for PTSD^10^, from a multi-ancestry GWAS from the Psychiatric Genomics Consortium, PGC).

Regarding polygenic scoring practices and as stated above, it is typical for researchers to separate samples into more ancestrally homogeneous groups prior to polygenic scoring analyses; Fig. 3 demonstrates why this is one sensible analytical choice. However, this is not always done. Some researchers are unaware of the extent to which ancestry can impact variant frequencies, and others may choose not to split samples because there is no clear choice regarding how multiple admixed and/or similar populations should be split. In all instances, proper use of principal components (PCs) or other methods of correcting for ancestry is critical. Further, Fig. 3 implies that there is no single recommendation for the number of PCs needed, given that PCs correlate with 1000Genomes populations (see Supplementary Fig. 3). Underscoring this point, Supplementary Fig. 4 shows the magnitude of correlations between 1000Genomes participants’ polygenic scores (for height, BMI, and schizophrenia) and the first 20 PCs (*N* = 2577; see Supplementary Fig. 5 for representative scatterplots and Methods for additional details). Two key conclusions can be drawn from these figures. It is important that multiple, sometimes non-consecutive, PCs are correlated with polygenic scores for each of these phenotypes. For example, for height polygenic scores constructed with GIANT summary statistics, it is primarily the first PCs (1–4) that are significantly correlated with polygenic scores, but non-consecutive and later PCs are also correlated (e.g., PCs 7 and 12, in this example). Second, results vary somewhat across a range of *p*-value thresholds (*p*_T_) used for constructing polygenic scores. These points underscore that using only a small (e.g., under 10) number of PCs may be inadequate for polygenic scoring analyses of mixed ancestry and admixed samples, and that careful inspection of data and plots is always needed.

### Putative correlations between worldwide phenotypes and PRSs

Finally, we turn to the most difficult question: what causes differences in polygenic scores, as currently calculated, among different populations? Differences could be real or artifactual (i.e., due to bias in data and/or methods), and five categories of explanations are listed below.

1. True differences due to drift
2. True differences due to selection
3. True differences in genetic effects due to environmental differences (gene-environment interactions)
4. Bias due to uncorrected population stratification in discovery and/or training samples
5. Bias due to discovery/training population data and/or polygenic scoring methods. Specifically, linkage disequilibrium (LD) structure and variant frequency are captured imperfectly with current methods (including genotyping and imputation), and they vary across populations, and currently available data resources are unequally representative of diverse worldwide populations.
6. Random error in the estimation of GWAS betas

Drift has been implicated as an explanation for population differences in polygenic scores among populations^32^, but others have reported that drift is insufficient to explain such differences^33^. Further, initial estimates of the strength of polygenic selection on height in European ancestry populations^33,37^ have recently been greatly reduced^30,31^, based on findings of uncorrected population stratification in summary statistics from the GIANT Consortium^30,31^. There is also disagreement about whether or not differences in average polygenic scores among populations might contribute to differences in phenotypic values among the same populations (which could also be due to environmental variation). Some have noted apparent positive correlations between average polygenic scores and phenotypes for BMI^34^, lupus^35^, and height as calculated using GIANT Consortium scores^33,36,37^. As described below, we include more data than used previously to address questions about potential correlations between worldwide height polygenic scores and height phenotypes.

**Fig. 4:**
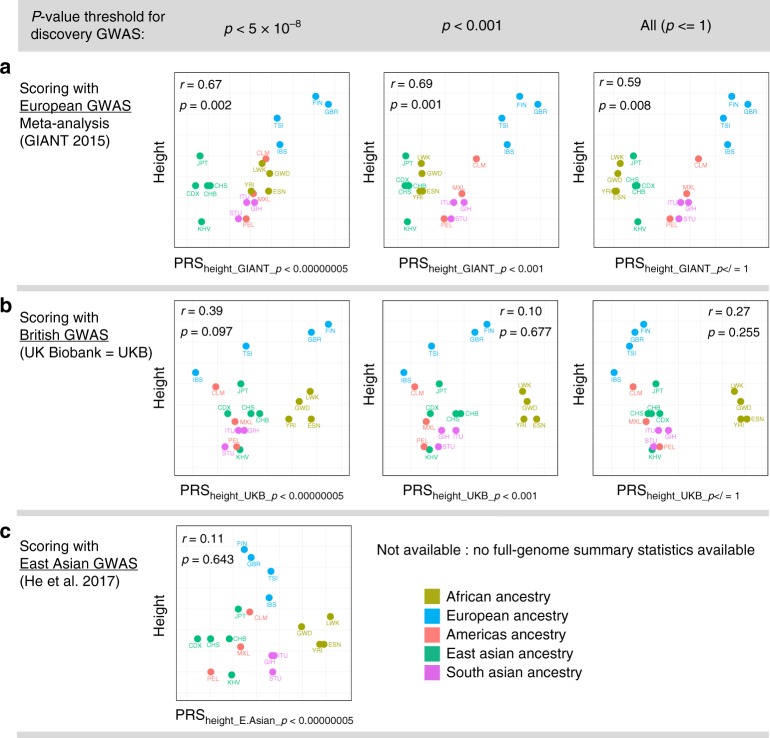
Scatterplots of height polygenic scores (*x*-axis) and phenotypic height (*y*-axis). Plots demonstrate that correlations between polygenic scores for height and height are not consistent across discovery GWAS. The *y*-values for height are the same for each plot and reflect average height of individuals in the country of origin for each population included. Average heights (*y*-axis) are from a different height GWAS used to construct polygenic scores (*x*-axis). Three different GWAS of height were used (i.e., three rows) with three different *p*-value thresholds (i.e., three columns) for the construction of polygenic scores. **a** GIANT-based polygenic scores for height. **b** UK Biobank-based polygenic scores for height. **c** East Asian based polygenic scores for height. The last two plots are missing because only genome-wide significant variants were available for the East Asian GWAS of height. *p* and *r* values for each plot are for correlation tests between polygenic scores for height (*x*-axis) and height (*y*-axis). GWAS=genome-wide association study, GIANT=Genetic Investigation of ANthropometric Trait, PRS=polygenic risk score, population abbreviations within scatterplots are those used by the 1000Genomes Consortium and are available in Supplementary Table 3

Using 1000Genomes data (as described in the Methods) and commonly used but different methodological choices in the construction of polygenic scores, we demonstrate that no simple conclusions can be drawn about polygenic scores and height for worldwide populations. In Fig. 4 we plot average polygenic scores for height of 1000Genomes populations on the *x*-axis, using three sources of weights for constructing scores (PRS = polygenic risk score):

- **4a** (top row) GIANT Consortium^18^ based scores: PRS_height_GIANT_
- **4b** (middle row) UKBiobank^44^ based scores from the NealeLab: PRS_height_UKBiobank_
- **4c** (bottom row) East Asian GWAS based scores^45^ from He et al: PRS_height_EastAsian_

On the *y*-axis, we plot average height for countries of origin for 1000Genomes populations, when available (see Methods for details and exclusions).

As shown in Fig. 4a, height phenotypes for worldwide populations (*y*-axis) are positively correlated with GIANT-based^18^ polygenic scores for height (*x*-axis), but not with UK-Biobank-based polygenic scores (**4b**) or East Asian GWAS based polygenic scores (**4c**). Polygenic scores constructed using only genome-wide significant variants from GIANT (top left) were positively correlated with height phenotypes (*r* = .67, *p* = .002), as were scores constructed using larger numbers of GIANT-based variants (e.g., all variants, top right, *r* = .59, *p* = .008). Results in **4b** and **4c** demonstrate that correlations (or lack of correlations) between height and polygenic scores for height are dependent on discovery GWAS. There are numerous reasons why polygenic scores differ between studies. However, recent findings suggest that correction for population stratification may not have been adequate in GIANT^30,31^, and therefore the positive correlations observed in **4a** could be partially due to uncorrected population stratification. The dependence of correlation estimates on discovery GWAS is further illustrated in **4c**, in which the point estimate for correlation between height and East Asian GWAS based polygenic scores for height is negative (*r* = −.11, *p* = .643). Power in discovery GWAS is also relevant, and greater confidence should be assigned to the results in **4a** and **4b** because both European ancestry discovery GWAS were adequately powered to detect hundreds of height loci, whereas the East Asian height GWAS was only adequately powered to detect 17 loci. Finally, methodological choices in polygenic score construction (see Fig. 3) must also be considered. The shape, dispersion, and even the ordering of distributions of polygenic scores for different 1000Genomes populations depends on polygenic score construction parameters, and would also necessarily result in different correlations with population phenotypes, and this is one additional reason why differences in polygenic scores among populations cannot be naively interpreted.

More research is needed to better understand the exact causes of differences in score distributions across populations and their putative relationships to phenotypes. Future research must also account for environmental effects on phenotypes, as well as variability in measurement validity and reliability across populations. Even for the relatively simple example of height (which is easily measured and for which major environmental influences are relatively well-understood) our analyses suggest that a great deal of caution should be used in drawing conclusions about polygenic score differences underling worldwide phenotypic differences, until data resources are significantly improved (i.e., well-powered GWAS in diverse populations), and until a deeper understanding of relevant population genetics principles has emerged. As discussed further below, even more caution will be required for other phenotypes such as psychiatric disorders.

## Discussion

As conversations about personalized medicine make their way to the general public, it is important to recognize the need to include under-represented populations in genetic studies. Among other concerns, the inclusion of participants representing diverse ancestries in research is imperative to ensure equitable benefit from scientific discoveries for diverse populations, and to prevent further increase in health disparities. Relevant to these longer-term objectives, our findings provide essential basic information about polygenic risk score usage among diverse populations, summarized in four key points. First, polygenic scoring studies have primarily been conducted in European and East Asian ancestry populations. Second, the performance of polygenic scores in non-European populations is generally poorer than performance in European ancestry samples, particularly for African ancestry samples. Third, polygenic scores for complex genetic phenotypes depend critically on the methods used to construct scores. Fourth, appropriate data resources are lacking to address most questions about putative differences in polygenic scores across worldwide populations. The straightforward, albeit expensive and time-consuming, solution to improving polygenic score performance across diverse populations is to create well-powered GWAS data resources for many different worldwide populations. However, the collection of very many samples from ancestrally disparate populations could be a poor use of resources, if all samples are underpowered. Perhaps the best approach is strategic collection of large samples from multiple populations, some of which may be ancestrally homogeneous, while others of which may be from specific admixed populations. This approach would afford cross-ancestry comparisons of more ancestrally homogeneous populations (which are easier to analyze) as well as much-needed method development for admixed populations (which are currently considered to be more difficult to analyze).

Our finding that the predictive power of polygenic risk scores is poorer in non-European populations, particularly among African ancestry individuals, is almost certainly due to the use of European ancestry training data, combined with the greater genetic diversity within many African populations, the phenotypic consequences of which are not well-captured by European ancestry GWAS. (Differences in heritability across populations for the same trait might also exist and could also impact the performance of polygenic scores in particular populations. Ultimately, the performance of a particular polygenic score, as applied in a particular population, should be evaluated with respect to the population-specific heritability for that phenotype). Indeed, the relative reduction of genetic diversity among European ancestry and other non-African populations (due to past population bottlenecks) is a natural limitation of European ancestry GWAS. In addition to the important goal of collecting more samples from more populations, there can also be improvements in the manner in which variant frequencies and linkage disequilibrium are handled in polygenic scoring studies. The results presented here provide benchmarks for the performance of polygenic scores in diverse populations, relative to performance in populations of European ancestry. This is important, because it not only informs power calculations for future research, but also highlights relative differences in predictive utility across diverse populations, which must be considered in public health and medical decisions regarding how and when to use polygenic risk scores.

Regarding expectations about polygenic score performance in non-European ancestry samples we note that polygenic scores for many complex genetic phenotypes are strongly correlated with global PCs, which highlights the critical importance of appropriate statistical methods for the analysis of genetic data from admixed populations, and caution in using imputed data from samples for which reference sequence datasets are lacking. Testing future polygenic scoring results for robustness to the inclusion of variable combinations of PCs will reduce the chances of spurious results (that are actually attributable to ancestry). In sum, the preponderance of genetic studies based on European ancestry samples has led to a situation in which polygenic scores are approximately one-third as informative for African ancestry individuals, as they are for European ancestry individuals. This is presumably true for commercially available tests as well; given these findings, consumers should be aware of the differential performance of tests across individuals.

Regarding scientific and public perception of polygenic scores, it is important to address apparent differences in polygenic score distributions across populations. Our findings suggest that it is currently not possible to know precisely the distribution of polygenic scores for non-European ancestry populations, for any complex genetic phenotype, for two major reasons: (1) score distributions depend critically on methodological choices in score constructions, and (2) data resources for most populations are currently inadequate. Further, as we have shown, the ordering of population distributions of polygenic scores varies under accepted methods of constructing these scores (i.e., using different *p*-value thresholds for variant inclusion in scores and using alternative discovery GWAS). Explanations for these differences are currently incomplete. Until vastly superior data resources are available—including large-scale GWAS in multiple globally representative populations—scientists are unlikely to reach consensus regarding the existence, nature, and exact causes of polygenic score differences among populations.

In our analysis of possible relationships between average phenotypes for worldwide populations and average polygenic scores for those populations, we chose to examine height because it is easily measured and because factors affecting height (e.g., nutrition) are also relatively easily quantified. In contrast, research on other variables such as weight, smoking status, psychological symptoms, and cognitive performance requires more careful control for environmental confounders (including variables like social status), which are often correlated with ancestry and therefore may also be correlated with global principal components and polygenic scores (as currently calculated). This means that confounding of environmental and genetic effects is likely. For example, social experiences such as being subjected to racism are prime candidates for confounding in genetic studies.

In closing, we emphasize the need to engage experts from other disciplines, such as social psychology and bioethics^46^, as geneticists attempt to characterize genetic effects on complex genetic phenotypes that are also affected by social factors (especially psychological and cognitive variables). This is necessary because societal influences including socioeconomic status and discrimination can powerfully influence these phenotypes^47^, and these causal social factors can co-vary with ancestry. In genetic research, there is a tendency not to include environmental influences on phenotypes (often due to resource constraints), but collaboration with disciplinary experts and increased attention from geneticists on gene-environment interplay (i.e., correlations and interactions) can address this problem which, we argue, will ultimately improve studies attempting to find genetic causes of human traits and behavior. Only then, with cautious and broadly-informed research, can the medical benefits of correctly interpreting polygenic variation within and among populations be realized.

## Methods

### Methods overview

This study has two major components. First, we analyzed results extracted from previous polygenic scoring studies in order to describe trends in polygenic scoring research. Second, we analyzed properties of polygenic scores as calculated for the 1000Genomes individuals, and compared polygenic scores to country-level information about height. This work received a notice of determination that it was not human subjects research from Stanford University.

### Part 1. Published polygenic scoring studies

See Supplementary Fig. 6 for a flowchart of study ascertainment, per PRISMA example. We first identified suitable studies via PubMed on 23 January 2018 using the following search terms: (Genome-Wide Association Stud* OR GWAS OR Genome Wide Association Stud*) and (polygenic risk score OR genetic risk score OR polygenic risk scor* OR genetic risk scor* OR risk profile scor* OR genomic profile). We sought to identify all polygenic scoring studies, of any complex genetic phenotype, from the first decade of polygenic scoring research. This yielded 1226 studies, 733 of which were polygenic scoring studies (see Fig. 1 and Supplementary Data 1).

We next identified studies that contained valid comparisons of the performance of polygenic scores in European ancestry participants and at least one other ancestry. Specifically, matched analyses (from two or more ancestry groups, from any given publication) had to use the same genotyping chip for all samples, the same weights for variants, the same algorithm for constructing polygenic scores, and the same methods of measuring phenotypes across all participants. Results from 26 studies met inclusion criteria, and there was minimal overlap in samples used (see Supplementary Data 2). From these studies we then extracted effect size metrics for each ancestry group. Effect size, refers to variance explained (the preferred metric) and to beta coefficients from regression, odds ratio (OR), or area under the curve (AUC), as available. Score performance for each analysis of a sample of non-European ancestry, was normalized to performance in the matched European ancestry sample from the same study, by dividing effect sizes (i.e., non-European ancestry/European ancestry). Odds ratios were first converted to log(OR). We multiplied values by 100 so that performance for each non-European ancestry sample could be expressed as a percentage of European ancestry performance, which was standardized to 100%. For example, the first polygenic scoring study of schizophrenia^12^ found that polygenic scores explained only 0.4% of phenotypic variance in an African ancestry sample, whereas 3.2% of phenotypic variance was explained in a matched European ancestry sample. Consequently, the value of 12.5% ((0.004/.032) × 100) is represented as the top-most yellow point in Fig. 2 (see schizophrenia and Purcell 2009 in the citation information for this point). By normalizing within-study effect sizes to European ancestry effect sizes, we were able to combine observations across phenotypes, and therefore to obtain general estimates of polygenic score performance across ancestry groups and complex genetic phenotypes.

### Part 2. Polygenic score properties for worldwide populations

For part 2, we used publicly available data from 1000Genomes^48^. Individual-level genotype data for 2577 individuals were downloaded from ftp-trace.ncbi.nih.gov/1000genomes/ftp/release/20130502/. Weights for constructing polygenic scores came from publicly available sources of GWAS results for height, body mass index (BMI), and schizophrenia^14,18,44,45^. For height we used GIANT summary statistics and UK Biobank statistics from the Neale Lab (version 1), which included 10 PCs as covariates, as calculated on all cleaned UK Biobank samples (i.e., including ancestrally diverse samples as well as the large majority of European ancestry samples). Data about average human height, for countries of origin for 1000Genomes populations, were downloaded from a pre-compiled table with male and female heights by country: https://en.wikipedia.org/wiki/List_of_average_human_height_worldwide.

Data preparation and analysis for 1000Genomes samples: The full 1000Genomes dataset was first filtered to include only bi-allelic single nucleotide polymorphisms (SNPs) with greater than 0.1% minor allele frequency. In order to calculate principal components across 1000Genomes genotypes, we used second generation PLINK^49^ to obtain variants in approximate linkage equilibrium, and we also removed the MHC region of chromosome 6 (25–35 Mb) and the large inversion region on chromosome 8 (7–13 Mb). We then calculated 20 PCs across all individuals.

In order to obtain weights for constructing polygenic scores, summary statistics files (i.e., GWAS results) were clumped to include only variants in approximate linkage equilibrium, using second generation PLINK^49,50^ and the following thresholds (varying the linkage disequilibrium *r*^2^ threshold): --clump-kb 500, --clump-p1 1, --clump-p2 1, --clump-r2 0.2 (and .05 and .01). 1000Genomes data were used as the source for linkage disequilibrium information for pruning all summary statistic files, and we varied the source populations to include all samples, and each of the major populations individually.

Second generation PLINK^49^ was used to construct polygenic scores for each phenotype for the 1000Genomes participants (*N* = 1940, see below for exclusions), using 13 thresholds, *p*_T_, as follows: *p* < 5 × 10^−8^, *p* < 1 × 10^−6^, *p* < 1 × 10^−4^, *p* < 1 × 10^−3^, *p* < 1 × 10^−2^, *p* < .05, *p* < .1, *p* < .2, *p* < .3, *p* < .4, *p* < .5, *p* < .75, *p* < = 1. The statistical package R^51^ was used for plotting and statistical analyses (*t*-tests, correlations). For correlations between polygenic scores and principal components, see Supplementary Data 3. Height phenotype data were downloaded from a compiled table of average heights, for males and females, by countries. Heights for males and females were averaged. Certain populations were excluded from the analysis of correlations between polygenic risk scores for height and height phenotypes for three reasons: Four populations were excluded due to lack of height phenotype data: Puerto Rican in Puerto Rico (PUR; *N* = 105), Bengali in Bangladesh (BEB; *N* = 86), Punjabi in Lahore, Pakistan (PJL; *N* = 96), Mende in Sierra Leone (MSL; *N* = 85). Two populations were excluded due to the combination of highly mixed country ancestry (impacting validity of height phenotype) and admixture of the 1000Genomes population (impacting variability in the polygenic scores for height): African Ancestry in Southwest US (ASW; *N* = 66), African Caribbean in Barbados (ACB; *N* = 96). One population was excluded due to the absence of a single European country of origin (needed for height phenotype information used in this report): Utah residents with Northern and Western European ancestry (CEU; *N* = 103). Details are given in Supplementary Table 1. All ethical regulations (including informed consent) were followed in the relevant studies, which provided the summary results used in this manuscript. This manuscript contains only secondary data analysis of publicly available data. No work with human participants was conducted, as determined by the Human Subjects review board at Stanford University.

## Supplementary information

Supplementary Information

Supplementary Dataset 1

Supplementary Dataset 2

Supplementary Dataset 3

Description of Additional Supplementary Files

## Data Availability

All data used in this report are publicly available as specified in this manuscript (with appropriate links) and/or are provided as Supplementary Data (i.e., modifiable.xlsx files).
