# Supplementary material for: Analysis of polygenic risk score usage and performance in diverse human populations
Source: Nat Commun. 2019 Jul 25;10:3328. doi: 10.1038/s41467-019-11112-0 (PMC6658471; doi:10.1038/s41467-019-11112-0)
Supplement: Supplementary file 1 — Supplementary Information [file 41467_2019_11112_MOESM1_ESM.pdf]

Supplementary Information

Analysis of polygenic risk score usage and performance in diverse human populations

Duncan, LE[a,], Shen, H[a], Gelaye, B[b], Joeri Meijsen[a], Ressler, KJ[c], Feldman, MW[d], Peterson, RE[e], Domingue, BW[f]

[a]Stanford University, Department of Psychiatry and Behavioral Sciences, 401 Quarry Road, Stanford, CA 94305, USA; [b]Harvard T.H. Chan School of Public Health, Department of Epidemiology, 667 Huntington Ave, Kresge 505, Boston, MA 02115, USA; [c]McLean Hospital, Harvard Medical School, Mailman Research Center, 115 Mill St. Belmont, MA 02478, USA; [d]Stanford University, Department of Biology, Herrin 478A, Stanford, CA 94305, USA; [e]Virginia Commonwealth University, Virginia Institute for Psychiatric and Behavioral Genetics, Department of Psychiatry, P.O. Box 980003, Richmond, VA 23298, USA; [f]Stanford University, Graduate School of Education, CERAS 510, Stanford CA, 94305, USA

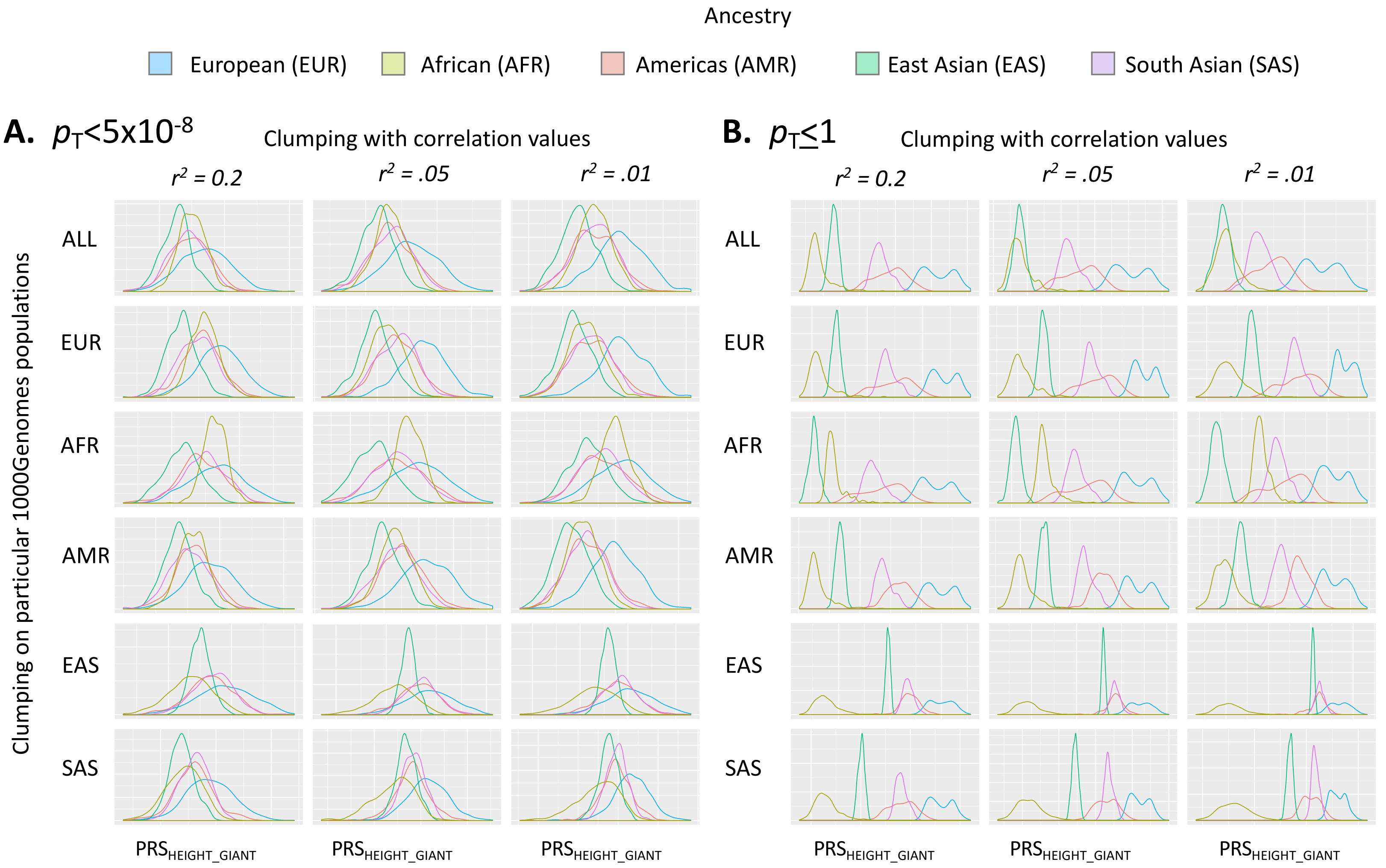

Supplementary Figure 1. Density plots of polygenic scores for 1000Genomes participants vary according to methodological choices in constructing polygenic scores (height GIANT). For all plots, weights were derived from the GIANT height GWAS and color-coding is according to 1000Genomes major populations. From left to right, r^2 values used in clumping were varied (r^2 = .2, .05, .01). From top to bottom, the 1000Genomes major population(s) used for clumping were varied (ALL 1000Genomes, EUR, AFR, AMR, EAS, SAS). A and B correspond to the p-value threshold applied to the summary statistics to specify variant inclusion/exclusion. **A)** p_t=genome-wide significant variants (p<5x10^{-8}); **B)** p_t= full genome variants (p</=1).

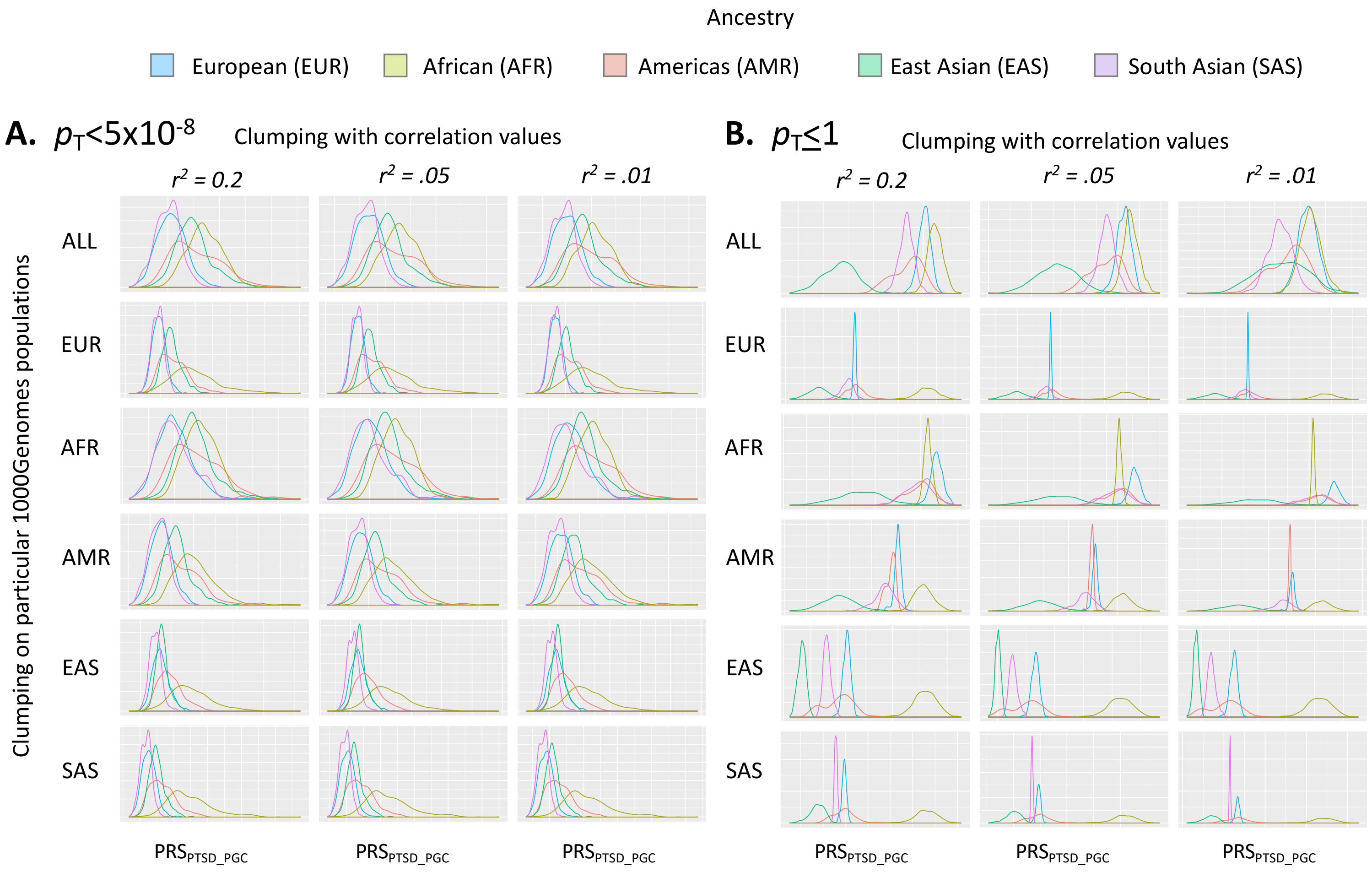

Supplementary Figure 2. Density plots of polygenic scores for 1000Genomes participants vary according to methodological choices in constructing polygenic scores (PGC PTSD). For all plots, weights were derived from the trans-ancestry Psychiatric Genomics Consortium post-traumatic stress disorder (PGC-PTSD) GWAS and color-coding is according to 1000Genomes major populations. From left to right, r^2 values used in clumping were varied (r^2 = .2, .05, .01). From top to bottom, the 1000Genomes major population(s) used for clumping were varied (ALL 1000Genomes, EUR, AFR, AMR, EAS, SAS). A and B correspond to the p-value threshold applied to the summary statistics to specify variant inclusion/exclusion. **A)** p_t=top variants (p<1x10^{-4}); **B)** p_t= full genome variants (p</=1).

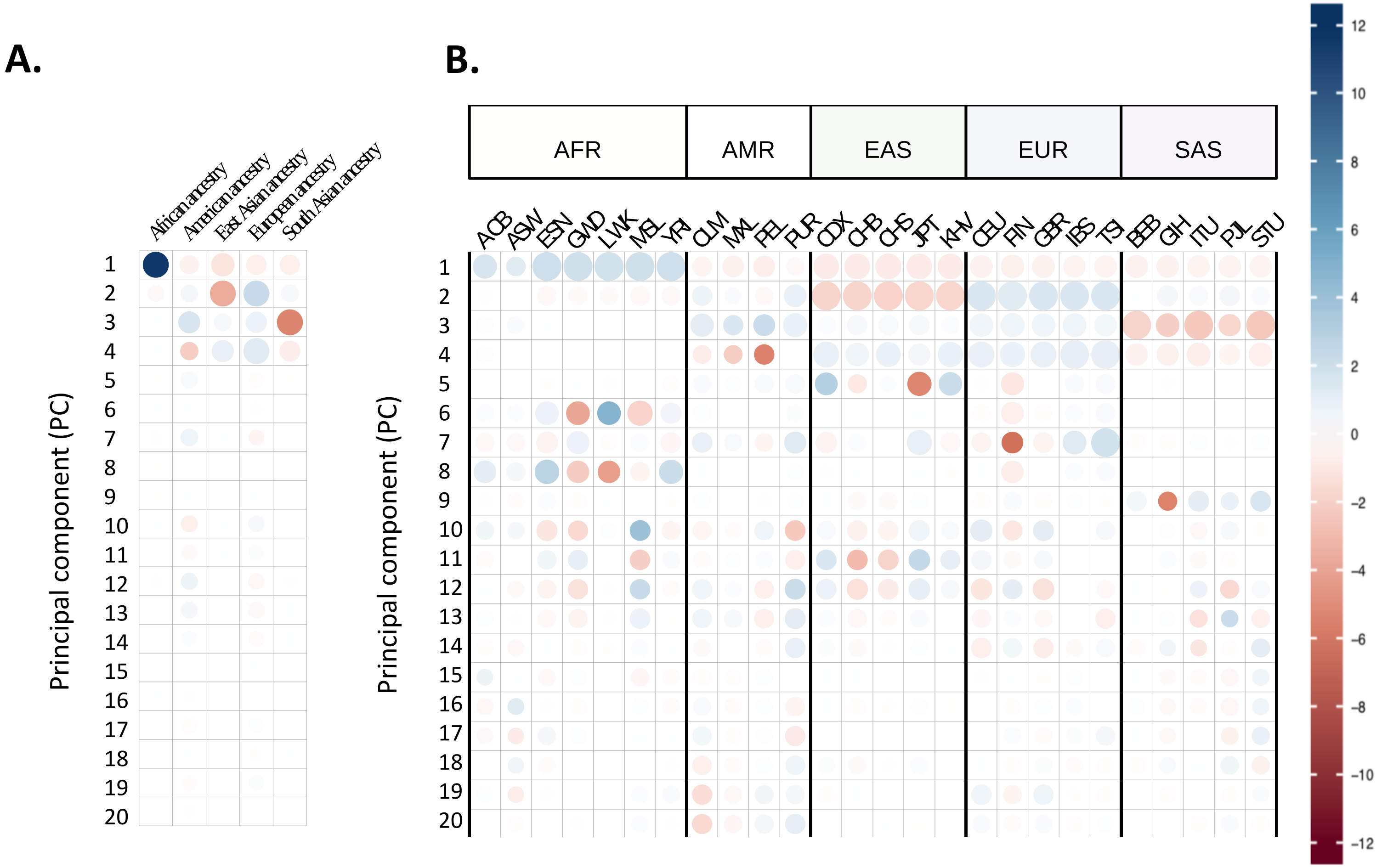

Supplementary Figure 3. Relationships between principal components (PCs) and population categories, for 1000Genomes participants. A) Five major 1000Genomes populations. B) Twenty-six sub-populations, ordered by major populations (1000Genomes standard abbreviations are used). Color denotes effect size (Cohen's d for each population as compared to all others).

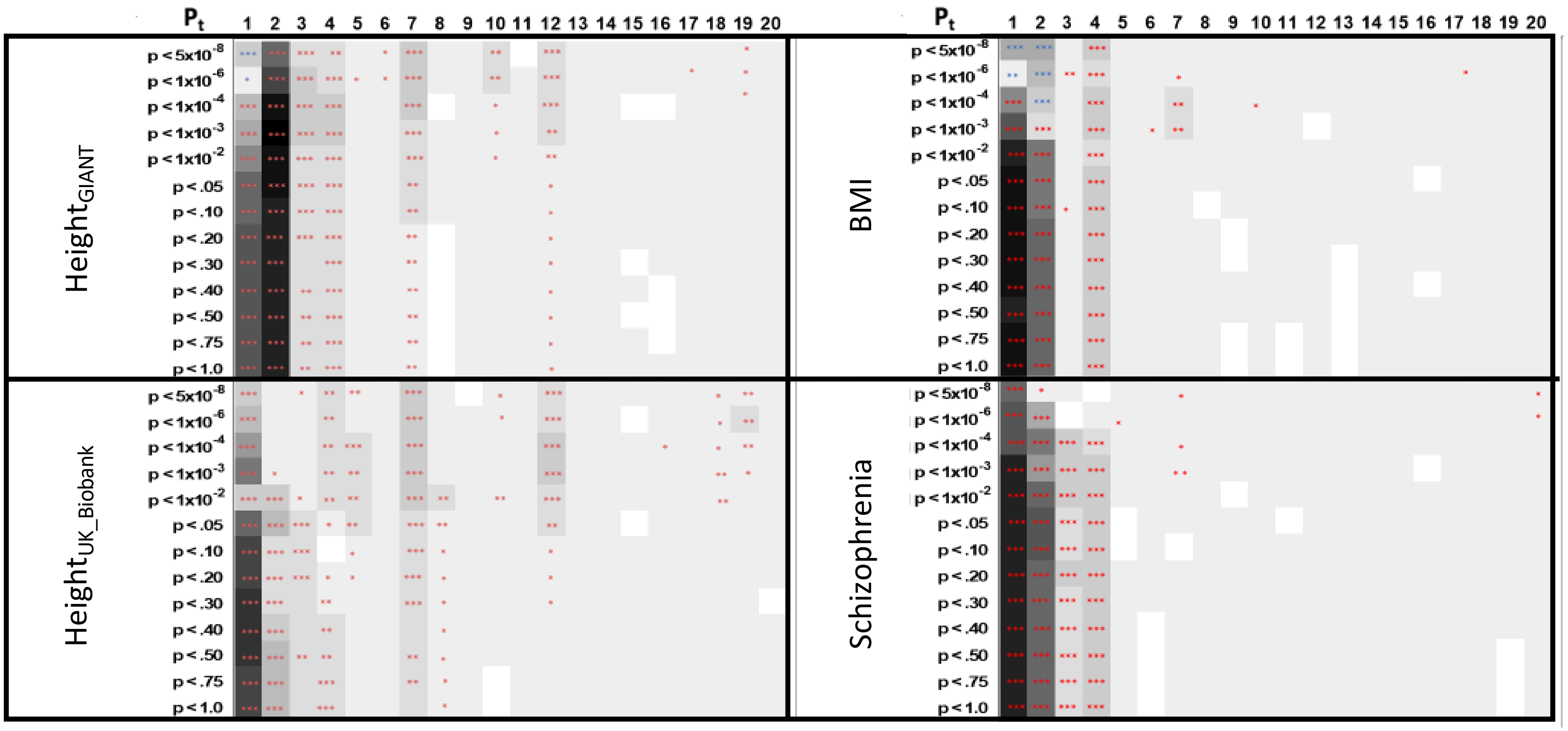

Absolute value of correlations between PCs and PRSs, *magnitude* (grayscale) and *significance* (asterisks)

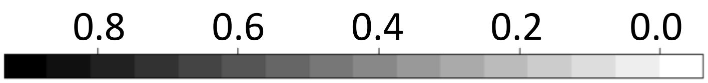

*	$p<.05$	* blue asterisks
**	$p<.005$	denote opposite direction
***	$p<5x10^{-5}$	correlations, within a given PC.

Supplementary Figure 4 Correlations between principal components (PCs) and polygenic scores for 1000Genomes participants, for four GWAS ($\text{height}_{\text{GIANT}}$, $\text{height}_{\text{UK\_Biobank}}$, body mass $\text{index}_{\text{GIANT}}$, and $\text{schizophrenia}_{\text{PGC}}$). For each phenotype, rows correspond to a range of p-values thresholds used to construct polygenic scores. See Supplemental Table 3 for exact correlations and p-values.

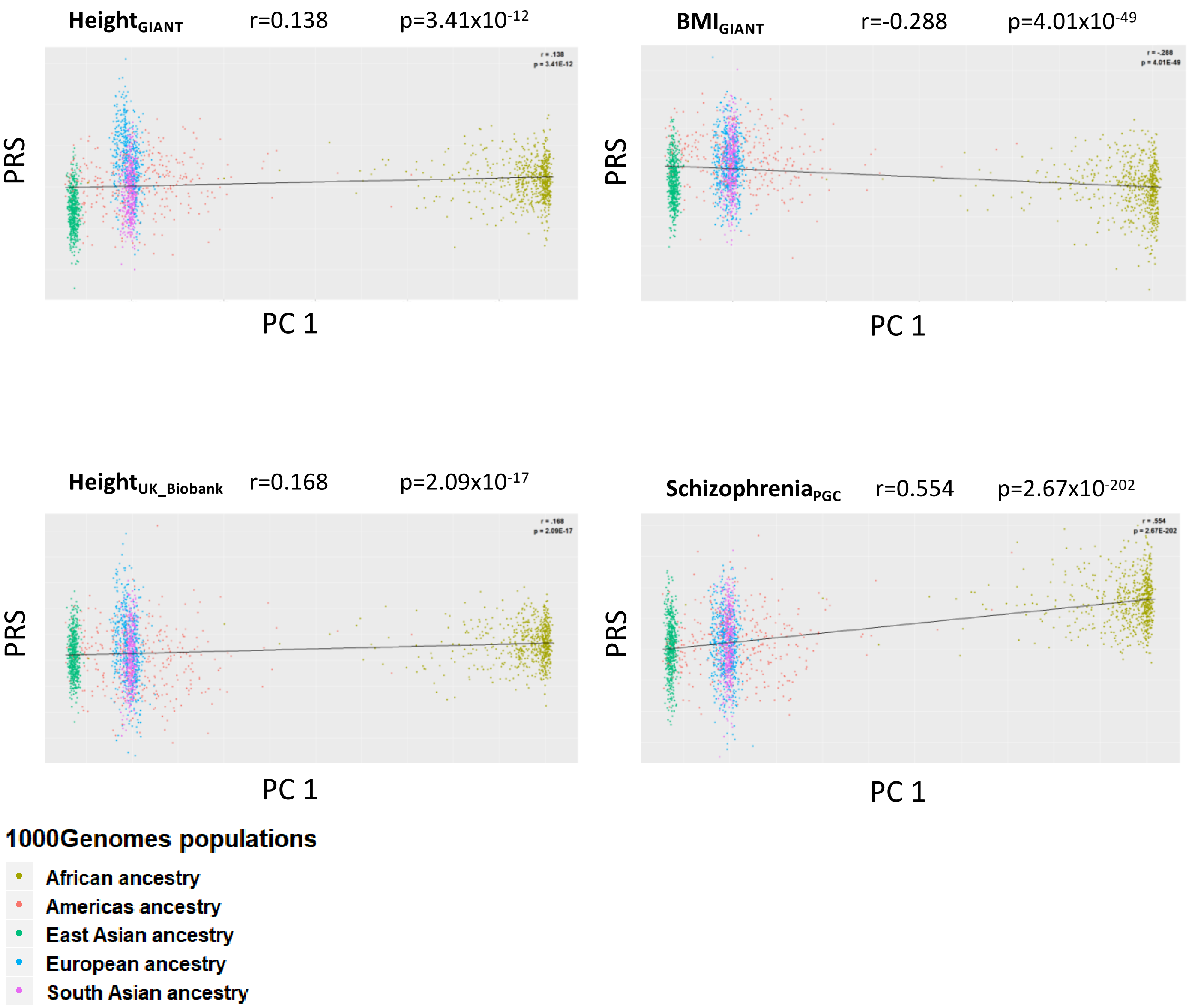

Supplementary Figure 5. Scatterplots of principal components (PCs) and polygenic scores for 1000Genomes participants. X-axis = scores on PC 1. Y-axis = polygenic risk score (PRS), using genome-wide significant variants to construct polygenic scores (p_T=5x10^{-8}).

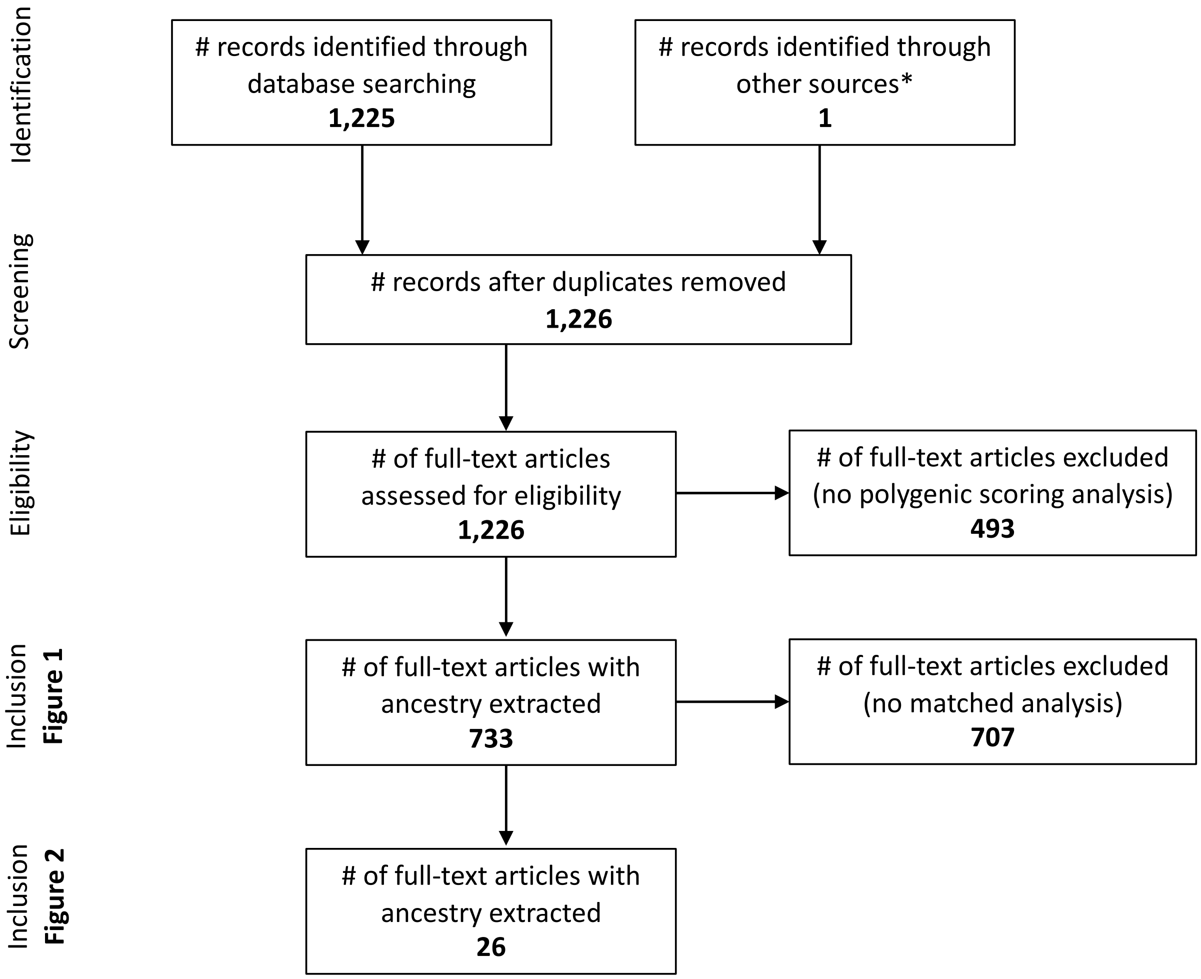

Supplementary Figure 6. Flowchart of study inclusion for Part 1, per PRISMA example. *the first empirical polygenic scoring study (2009 Purcell et al.) did not appear in the search, apparently due to keyword mismatch issues.

Population	Total	Country
Chinese Dai in Xishuangbanna, China (CDX)	99	China
Han Chinese in Bejing, China (CHB)	106	China
Japanese in Tokyo, Japan (JPT)	105	Japan
Kinh in Ho Chi Minh City, Vietnam (KHV)	101	Vietnam
Southern Han Chinese, China (CHS)	112	China
Total East Asian Ancestry (ASN)	523	
Total included East Asian Ancestry (ASN)	523	
Bengali in Bangladesh (BEB)	86	remove[1]
Gujarati Indian in Houston, TX (GIH)	106	India
Indian Telugu in the UK (ITU)	103	India
Punjabi in Lahore, Pakistan (PJL)	96	remove[1]
Sri Lankan Tamil in the UK (STU)	103	Sri Lanka
Total South Asian Ancestry (SAN)	494	
Total included South Asian Ancestry (SAN)	312	
African Ancestry in Southwest US (ASW)	66	remove[2]
African Caribbean in Barbados (ACB)	96	remove[2]
Esan in Nigeria (ESN)	99	Nigeria
Gambian in Western Division, The Gambia (GWD)	113	Gambia
Luhya in Webuye, Kenya (LWK)	116	Kenya
Mende in Sierra Leone (MSL)	85	remove[1]
Yoruba in Ibadan, Nigeria (YRI)	116	Nigeria
Total African Ancestry (AFR)	691	
Total included African Ancestry (AFR)	444	
British in England and Scotland (GBR)	94	UK
Finnish in Finland (FIN)	100	Finland
Iberian populations in Spain (IBS)	107	Spain
Toscani in Italy (TSI)	110	Italy
Utah residents with Northern and Western European ancestry (CEU)	103	remove[3]
Total European Ancestry (EUR)	514	
Total included European Ancestry (EUR)	411	
Colombian in Medellin, Colombia (CLM)	95	Colombia
Mexican Ancestry in Los Angeles, California (MXL)	69	Mexico
Peruvian in Lima, Peru (PEL)	86	Peru
Puerto Rican in Puerto Rico (PUR)	105	remove[1]
Total Americas Ancestry (AMR)	355	
Total included Americas Ancestry (AMR)	250	
Total	2577	
Total included	1940	
Percent included	75.3	

Supplementary Table 1. 1000Genomes samples (from http://www.1000genomes.org/about on 8/12/14). [1]Removed due to missing height phenotype data; [2]Removed due to combination of highly mixed country ancestry (impacting validity of height phenotype) and admixture of the 1000Genomes population (impacting variability in the polygenic scores for height); [3]Removed due to the absence of a single European country of origin.
