# Supplementary material for: Analysis of polygenic risk score usage and performance in diverse human populations
Source: Nat Commun. 2019 Jul 25;10:3328. doi: 10.1038/s41467-019-11112-0 (PMC6658471; doi:10.1038/s41467-019-11112-0)
Supplement: Supplementary file 5 — Description of Additional Supplementary Files [file 41467_2019_11112_MOESM5_ESM.docx]

**Title:** Supplementary Data 1.
**Description:** 733 studies meeting inclusion criteria for Figure 1.

**Title:** Supplementary Data 2.
**Description:** 26 studies meeting inclusion criteria for Figure 2.

**Title:** Supplementary Data 3.
**Description:** Correlations between polygenic scores and principal components (r and p values).
